# Infectious crystalline keratitis induced by Citrobacter

**DOI:** 10.3205/oc000182

**Published:** 2021-05-17

**Authors:** Zeba Khanam, Gaganjeet Singh Gujral, Shariq Wadood Khan

**Affiliations:** 1 Institute of Ophthalmology, J. N. Medical College, Aligarh Muslim University, Aligarh, Uttar Pradesh, India; 2 Department of Microbiology, J. N. Medical College, Aligarh Muslim University, Aligarh, Uttar Pradesh, India

**Keywords:** microbial keratitis, crystalline keratopathy, Citrobacter, levofloxacin

## Abstract

**Background:** Microbial keratitis is an important cause of ocular morbidity, with emerging organisms and drug resistance posing a real threat to vision of patients.

**Case presentation:** A 30-year-old female presented with infective keratitis in the left eye. She had been using rose nectar as home remedy for her ailment. With no improvement in her symptoms, she presented to the eye emergency department, where she was started on empirical therapy with moxifloxacin, which was shifted to levofloxacin eye drops after the antimicrobial susceptibility test results came in. Microbiological examination revealed infection with rare gram-negative bacilli *Citrobacter koseri*. The patient responded well to the treatment with 1.5% levofloxacin eye drops and her vision improved from 20/120 to 20/30 over a course of 3 months.

**Conclusion:** Treatment of microbial keratitis with those antibiotics that the organism is most sensitive to is of paramount importance today, where we often find patients on a cocktail of eye drops, which leads to further resistance and vision deterioration. Culturing of cornea scrapings and antimicrobial susceptibility testing of the isolated organism is now the standard guideline to be followed in the investigation of microbial keratitis.

## Background

The *Citrobacter* species are ubiquitous gram-negative bacilli, rarely causing infection in humans. While the organism usually infects immunocompromised hosts, patients can contract *Citrobacter* without underlying disease [1]. We report this rare case of microbial keratitis caused by *Citrobacter koseri*, and its approach in diagnosis as well as management.

## Case description

A 30-year-old female presented to the outpatient department with complaints of redness, pain and watering in the left eye for 1 week. It was associated with diminution of vision and photophobia. There was no history of ocular trauma or any systemic illness preceding the symptoms. The patient was non-diabetic.

The patient reported that her symptoms started in the morning after waking up. She instilled rose nectar in her left eye, about two times a day for about 1 week. As there was no relief in her symptoms, she reported to our eye emergency department.

**Figure 1:**
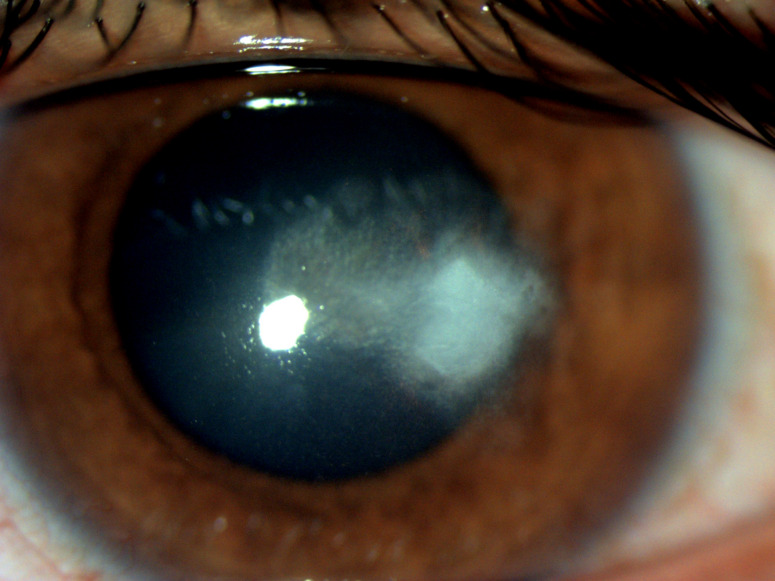
Slit-lamp examination of the left eye at initial visit shows white, branching, needle-like, crystalline corneal stromal infiltrate

**Figure 2:**
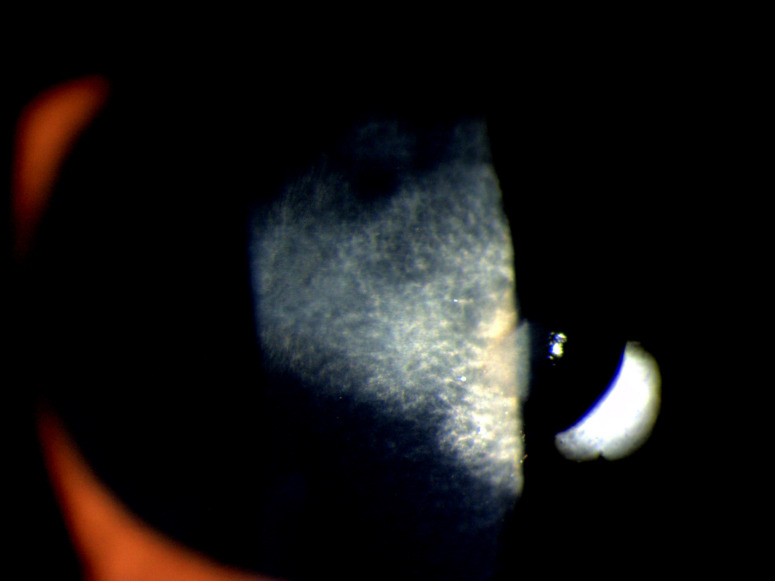
Magnified view of the corneal crystalline infiltrates

The ophthalmic examination revealed visual acuity (VA) of 20/20 in the right eye (RE) and 20/120 in the left eye (LE). Slit-lamp biomicroscopy of the RE showed quiet eye with clear cornea and normal pupillary reaction. The LE showed superficially congested conjunctiva and central corneal ulcer with infiltrates present in the corneal stroma (Figure 1 (Fig. 1)). There were multiple, coarse, white needle-like opacities reaching deep into the stromal interface (Figure 2 (Fig. 2)). The pupil was of normal size with normal reaction to light, and the lens was clear. There were no signs of inflammation in the anterior chamber. The patient’s intraocular pressure was 16 mm Hg, and 18 mm Hg in the RE and LE respectively. The fundus evaluation of both eyes was within normal limits.

Corneal scraping of the LE was done with blade no. 15 under aseptic precautions from the base and margins of the ulcer and sent for gram staining, potassium hydroxide (KOH) mount, as well as bacterial culture and antimicrobial susceptibility testing. The patient was started on empirical therapy with moxifloxacin 0.5% eye drops 2-hourly and cyclopentolate 1% eye drops thrice a day in the left eye.

Microbiological examination revealed a gram-negative bacillus that was seen to rapidly ferment lactose and produce indole. The KOH mount was negative. This was consistent with growth of *Citrobacter koseri*. Antimicrobial susceptibility testing was performed by using the Kirby-Bauer disk diffusion method. The reading was performed by measuring the diameter of the inhibiting zone around the disk, in agreement with the Clinical & Laboratory Standards Institute (CLSI) 2020. The organism was sensitive to levofloxacin with resistance to vancomycin, amikacin, gentamicin and ceftazidime. The patient was switched to 1.5% levofloxacin 5 eye drops 2-hourly in the left eye after 48 hours when the culture and sensitivity reports were made. Cyclopentolate eye drops was continued thrice daily.

After 1 week of initiating the levofloxacin therapy, the symptoms of pain and watering had decreased. VA was 20/80. Slit-lamp biomicroscopy showed infiltrates present in the posterior stroma of the cornea with decreased conjunctival congestion. The patient was continued on levofloxacin eye drops with stopping of cyclopentolate eye drops. Carboxymethylcellulose 0.5% eye drops were added four times a day for lubrication and increased comfort.

**Figure 3:**
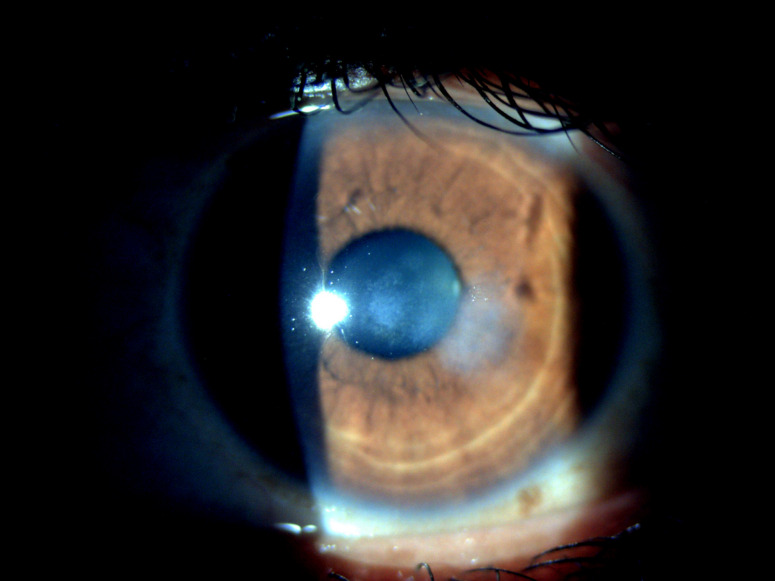
Slit-lamp examination of the left eye at 3 months showing resolved lesion with decreased infiltration

The patient did well over the course of 3 months. Her VA in the LE improved to 20/30. There was a decrease in the size and depth of infiltration with formation of the resolved scar (Figure 3 (Fig. 3)).

## Discussion

Microbial keratitis is a frequent cause of sight loss which leads to substantial morbidity in the developing parts of the world [1]. It is characterized by a corneal epithelial defect with underlying stromal inflammation caused by replicating microorganisms. The initial signs and symptoms of corneal ulcer depend on the virulence of the bacteria, and often start with eye pain, photophobia, ciliary congestion and decreased visual acuity.

*Citrobacter* are rare and emerging bacteria causing keratitis in the developing world. *Citrobacter koseri* (previously known as *Citrobacter diversus*) is a facultative anaerobic gram-negative bacillus of the family *Enterobacteriaceae*. The *Citrobacter* species are occasional inhabitants of human and animal intestines and of soil, water, sewage, and food [2]. Infectious crystalline keratopathy (ICK) is a minimally inflammatory infection as compared with typical microbial keratitis which is associated with acute suppurative reaction, due to indolent nature of the micro-organisms and concurrent use of the corticosteroids. In ICK, colonies of microorganisms produce branching crystalline opacities within the corneal stroma.

Most cases of infectious crystalline keratopathy are associated with gram-positive bacteria, especially the *Streptococcus* species (α-hemolytic *Streptococcus* is the most common cause). Gram-negative bacteria have rarely been associated with infectious crystalline keratopathy [3]. 

A review of all the patients diagnosed with ICK at Cullen Eye Institute between 1978 and 1995 revealed 18 cases. Of these, 10 cases had gram-positive cocci, five had gram-negative rods (*Acinetobacter Iwoffii*, *Citrobacter koseri*, *Enterobacter aerogenes*, *Pseudomonas aeruginosa*, and *Stenotrophomonas maltophilia*), and three had positive cultures for fungi. There was no significant difference in the rates of predisposing factors, the clinical appearance or the final visual outcome.

In our case, the patient presented with diminution of vision, redness and watering, and on examination there was a minimal inflammatory reaction with infiltrates present on the stroma. On follow-up, branching crystalline opacities were seen in the corneal stroma.

## Conclusions

We conclude that gram-negative bacteria can cause infectious crystalline keratopathy but have no distinguishing features from infectious crystalline keratopathy caused by *streptococci* and other gram-positive bacteria. Corneal scrapings for staining and antimicrobial susceptibility test results to guide appropriate antimicrobial therapy are necessary for appropriate diagnosis. Antimicrobial susceptibility test results are extremely useful as drug resistance to routinely used anti-microbial drugs is common and we could end with non-responsive cases with the empirical treatment [4].

## Notes

### Informed consent

Informed consent has been obtained from the patient for the publication of this case report.

### Competing interests

The authors declare that they have no competing interests.
